# A scoping review of international policy responses to mental health recovery during the COVID-19 pandemic

**DOI:** 10.1186/s12961-020-00652-3

**Published:** 2021-04-06

**Authors:** Claire McCartan, Tomas Adell, Julie Cameron, Gavin Davidson, Lee Knifton, Shari McDaid, Ciaran Mulholland

**Affiliations:** 1 Centre for Evidence and Social Innovation, Queen’s University Belfast, Belfast, United Kingdom; 2 Department of Health Northern Ireland, Mental Health Capacity Unit, Belfast, United Kingdom; 3 Mental Health Foundation, Glasgow, United Kingdom

**Keywords:** Mental health, COVID-19, Coronavirus, Mental health policy, Digital healthcare, At-risk groups, Social determinants

## Abstract

The COVID-19 pandemic has affected people’s physical and mental health. Quarantine and other lockdown measures have altered people’s daily lives; levels of anxiety, depression, substance use, self-harm and suicide ideation have increased. This commentary assesses how international governments, agencies and organisations are responding to the challenge of the mental health impact of COVID-19 with the aim of informing the ongoing policy and service responses needed in the immediate and longer term. It identifies some of the key themes emerging from the literature, recognises at-risk populations and highlights opportunities for innovation within mental health services, focusing on the published academic literature, international health ministry websites and other relevant international organisations beyond the United Kingdom and Ireland. COVID-19 has challenged, and may have permanently changed, mental health services. It has highlighted and exacerbated pre-existing pressures and inequities. Many decision-makers consider this an opportunity to transform mental health care, and tackling the social determinants of mental health and engaging in prevention will be a necessary part of such transformation. Better data collection, modelling and sharing will enhance policy and service development. The crisis provides opportunities to build on positive innovations: the adaptability and flexibility of community-based care; drawing on lived experience in the design, development and monitoring of services; interagency collaboration; accelerating digital healthcare; and connecting physical and mental health.

## Background

The coronavirus disease 2019 (COVID-19) pandemic first detected in China in December 2019 has had a substantial impact on people’s physical and mental health. In October 2020, over 37 million cases have been reported including almost 1.1 million deaths [1]. Epidemics are detrimental to public mental health [2] and contemporaneous data from national population surveys has identified a surge in mental health problems [3–5]. An increase in the number and severity of mental health problems is anticipated and likely to have long-term impact [6]. Equally, the world economy has been traumatised; it is estimated that an additional 400–600 million people will be forced into poverty because of COVID-19 [7].

Major economies have tried to mitigate the fiscal shock using a range of measures such as tax deferrals, income and loan subsidies, but unemployment is expected to rise significantly. Economic forecasts suggest the global economy is heading for the deepest recession since the Great Depression (1929–1933) and the Organisation for Economic Co-operation and Development (OECD) projects that the United Kingdom economy will suffer some of the worst damage globally [8]. Deprivation and the coronavirus are associated—the most deprived areas of England had more than twice the death rate than the least deprived areas [9]. Globally, people experiencing multidimensional poverty are particularly susceptible to contracting the virus [10]. Existing socioeconomic, health and other inequalities are likely to be compounded by the challenges ahead.

### Pre-COVID-19 mental health inequalities

The social determinants of mental health are well established [11]. Social inequality is a risk factor for mental ill health and those experiencing poverty and other inequities suffer disproportionately [11]. Unemployment, precarious employment, poor working conditions and low income are routinely linked to psychological distress [12, 13]. Neighbourhood deprivation is associated with poorer mental health, higher levels of psychiatric prescriptions and hospitalisation for mental disorders [14]. The impacts of poverty on food insecurity, diet and nutrition have also been linked to poorer mental health [15]. There is inequality in the allocation of funding to mental health services, typically receiving less funding than physical healthcare. Globally, mental health has lacked significant investment with an average spend of only 2% of health budgets [6] although the range varies greatly between low- and middle-income countries (LMICs) and high-income countries, reflecting not only economic wealth but also different levels of income inequality, healthcare funding models and the relative prioritisation of mental health (in France, for example, 15.0% of the total healthcare budget is dedicated to mental health, and less than 0.05% in the United States) [16]. There are also variations in how budgets are allocated within mental health services, with the majority of expenditure typically directed to mental hospitals in LMICs (more than 80% compared to less than 43% in high-income countries) [16].

### Impact of COVID-19 on mental health

There is emerging evidence that the pandemic is having a negative effect on mental health at a population level [5]. The impact of social isolation, bereavement, and an increase in negative coping skills, including substance use, may also have long-term consequences. A reduction in the availability and breadth of mental health services has had an impact on people with existing mental health problems and those who are experiencing new difficulties with their mental health during the pandemic. While demand for face-to-face services significantly decreased [17] and some people were more reluctant to report or seek help for mental health problems during lockdown [18], pressure on digital and telehealth service contact increased [19–21]. However, this increase in effective and scalable digital and online services is in danger of alienating some at-risk and socially excluded populations [6].

The aim of this scoping review is to assess how international governments, agencies and organisations have responded to the challenge of the mental health impact of COVID-19 in order to identify common themes and innovative developments to further inform the ongoing immediate and longer-term policy and service responses that are needed.

## Methods

Key databases (MEDLINE, Embase, PsycInfo), worldwide health department/ministry webpages and a general web-based search was conducted using variations of (COVID-19 AND mental health policy) in June 2020. A total of 78 documents were included in the policy review including peer-reviewed journal articles (35), governmental/policy briefing reports (21), webpages (19), newspaper articles (2) and a webinar (1). Only papers in English were retrieved in the search which may have skewed the results towards United Kingdom-based or a global or European focus. A number of articles also reported country-specific COVID-19-related research or policy including Aotearoa New Zealand, Australia, China, Finland, India, Iran, Italy, Lebanon, Spain and the United States. Thematic analysis identified some of the common cross-cutting key themes in this broad range of literature.

### International mental health planning and recovery

#### International co-operation

The WHO and the United Nations (UN) have produced policy briefings on mental health recovery. The OECD is compiling data, analysis and recommendations on COVID-19 policy responses [22] and professional bodies and third sector organisations have also generated policy responses and guidance [23, 24]. A growing number of fast-tracked journal articles are being published identifying mental health care priorities as a result of the pandemic [25–28].

The UN’s policy brief [6] calls for a ‘whole-of-society’ approach to promote, protect and care for mental health that includes widespread availability of emergency mental health and psychosocial support (MHPSS). It appeals for additional investment in interventions delivered remotely and uninterrupted in-person care for severe mental health conditions ensuring continuity of care. It also identifies COVID-19 as an opportunity to transform mental health services as part of universal healthcare, towards services that are centred around communities and that consult and involve people with lived experience in the design, delivery and monitoring of care.

WHO’s Inter-Agency Standing Committee (IASC) Reference Group on MHPSS in Emergency Settings produced an interim briefing note in February 2020 [29]. This gives specific guidance for MHPSS in emergency settings in order to mobilise an interagency response. It recommends that multiple levels of interventions are integrated within outbreak response activities using a series of overarching principles including identifying at-risk populations; mapping current services and interagency collaboration; building on community resourcefulness; and adopting a long-term perspective to strengthen mental health, social care and social welfare.

Calls for international co-operation in mental health strategies have also been voiced. The European Union (EU) has committed to publishing a mental health strategy by the end of 2020 and this is expected to focus on co-ordinating data collection and sharing best practice rather than increasing EU healthcare capability. A joint taskforce has been established by the WHO/Europe and the Central European Initiative to respond to COVID-19 across the region. It is difficult to make clear comparisons in how individual countries have been affected because of a lack of, and consistency in, data collection. In Finland, for example, early research results have found that the pandemic has had a smaller impact on psychological well-being than originally feared [30]. However, in Belgium, almost all face-to-face therapies were cancelled and 60% of mental health service users in the Netherlands reported partial or entire suspension of their treatment, with 80% unable to access daily care [31]. The medium- to long-term impact of the suspension of services is unclear.

#### Individual country-level responses

Two countries that have had some of lowest death rates, by global comparison, have issued mental health response plans, Australia [32] and New Zealand [33]. Lower mortality rates have arguably been associated with more effective elimination strategies including border controls, mass testing and technology rather than the mitigation measures aimed at flattening the curve of infection [34]. It may be that lower mortality has allowed emergency service planning and response to expand their focus on the wider psychological impact of the pandemic. New Zealand is the only nation to produce a dedicated mental health recovery plan. It details five areas of focus that consider: the social determinants of health; the central role of communities and interagency collaboration to respond and aid recovery; and include practical measures that promote self-care, strengthen primary mental health/addiction care; and emphasise the importance of collecting good quality data and enhancing specialist services (particularly for at-risk populations). These themes are evident in the growing literature.

A number of countries have used a common framework to develop action plans. The WHO Eastern Mediterranean Region is implementing activities to address MHPSS as part of their national response including the expansion of dedicated phone and online help and support (e.g. Afghanistan, Egypt, Iran, Iraq, Palestine). In co-operation with WHO (which has a specific focus on MHPSS in emergencies) and the United Nations Children’s Fund (UNICEF), the Lebanese Government has developed their action plan based on local needs, their health system and national mental health strategy, providing psychological first aid and emotional crisis training for healthcare staff.

Other countries have developed resources that could be extended to form part of a mental health recovery plan. In China, online psychological services are providing free 24-hour care. Self-help interventions including online cognitive behavioural therapy (CBT) have been used and there has been an increase in online mental health education on social media. Artificial intelligence (AI) is identifying individuals at risk of suicide by monitoring messages posted on Weibo (the Chinese version of Twitter) [35]. The Netherlands has established a single online portal to promote mental health led by the ARQ National Psychotrauma Centre (ARQ) Centre of Expertise on Disasters and Crises. The portal provides information for professionals and the general public directing queries to local and regional aid, care and service providers. The portal will be active for at least 2 years. ARQ also contributes to the development of measuring instruments (including a resilience monitor) and implementation research enabling a unique overview and an outcomes-focused approach.

## Key themes emerging from the literature

### Tackling the social determinants of health

Mental health services have had significant underinvestment over decades and adequate resourcing will be necessary to help an effective and sustainable recovery. While additional resourcing of mental health care is required, a more fundamental change to redress social inequality is crucial. Tackling social inequalities is key and the debate about a basic income guarantee has been put into sharp focus. Many countries have provided one-off payments to families and government-guaranteed income subsidies and furlough schemes to support workers’ incomes. A basic income guarantee could contribute to supporting at-risk populations and help protect against financial problems that could be detrimental to well-being. Evidence from Finland’s recent 2-year universal income pilot was positive, reporting better financial well-being, mental health and cognitive functioning and feeling more positive about the future compared to the control group [36].

### New ways of working

The pandemic has forced an acceleration of new and different ways of working, including the growth of digital mental health support.

#### Digital healthcare

The Director of Mental Health Europe, Claudia Marinetti, described the growth of mental health online professional care during the pandemic as the ‘silver lining’ in the absence of other care options. While careful consideration is required, digital technology has exciting potential to transform services and increase capacity, but it cannot always replace the importance of physical interactions to deliver certain aspects of healthcare, particularly with socially and digitally excluded groups [37, 38].

#### Building stronger communities

Similarly, the galvanisation of communities to support the vulnerable has been evident throughout the pandemic—there have been huge responses to government appeals for volunteers and spontaneous action of local communities providing practical and social support. Voluntary services cannot replace funded healthcare but these community responses have highlighted the importance of creating a sense of belonging and connection to an identity/place/community, central to increasing social capital and empowering communities. The capacity that communities have shown during the crisis could be an important factor in reforming public services and the prevention of mental health problems [39, 40].

#### Co-production and lived experience

A common theme in both the immediate response to the emergency and planning for the medium- to long-term recovery is the importance of involving people with lived experience, their carers and family members in the design, delivery and monitoring of services. A number of the policy and government responses have placed this as a central tenet of transforming care [6, 29, 32, 33]. How this can be implemented meaningfully requires careful consideration and should include effective ways to measure related outcomes. These could include identifying clear roles for involving consumers and carers [32] and good quality data collection [33].

#### Improving data quality and modelling

Data is key to understanding emerging needs, mapping services and planning a comprehensive service response. Many countries have already well-established health and social care monitoring systems that cut across different agencies (see for example, Norway and Finland). Both Australia’s and New Zealand’s pandemic response plans highlight data collection as an important enabler. Having capacity within health systems to conduct statistical modelling can improve current delivery.

#### Increasing physical activity

There is strong evidence of the benefits of physical activity to both prevent and treat mental ill health [41] and COVID-19 recovery presents an opportunity to reinforce this message and transform public transport to help promote more active and sustainable ways to commute/travel. Levels of physical activity have increased in the general population during the pandemic as governments reinforce the public health messaging about the importance of physical activity to support good mental and physical well-being. Changes to promote greener commuting have been fast-tracked with expansions of cycle and walk schemes. How we build on these new habits could be crucial in supporting well-being.

#### Re-evaluating key workers and ‘re-productive work’

During the pandemic, work and economic opportunities for women have been impacted as the burden of child and family care has largely fallen to female family members, with knock-on effects to their mental health [42, 43]. Similarly, calls for the re-evaluation of low-paid key workers (who are disproportionately female) have been made; whether this recalibration will be sustained beyond the current crisis is unclear.

#### Focus on prevention, promotion and help-seeking

There has been a new and concentrated focus on self-care and self-help during the pandemic and greater promotion of prevention and early intervention for mental health support. Effective self-help interventions [44, 45] could help reduce a spike in need for secondary/tertiary mental health services.

### At-risk groups

We know that mental health problems are not usually experienced in isolation and co-exist within different social, political and economic contexts. Recent research conducted by the Mental Health Foundation has highlighted some of these complex issues in relation to COVID-19 [46] and identified key at-risk groups including ethnic minorities, people with disabilities, single parents, people with pre-existing mental health problems, LGBT+, victims of abuse and people living in poverty and unemployment. Ethnic minorities have been disproportionately affected by COVID-19 and are at increased risk of death [47]. There are also lessons from how COVID-19 has affected at-risk and socially excluded populations that could help inform future healthcare delivery. Many of these groups had complex health needs prior to the pandemic and COVID-19 has restricted their access to healthcare and other essential services. COVID-19 has highlighted the health inequalities of these populations; meeting the health and social care needs of marginalised groups must include addressing the difficulties delivering supportive care in times of crisis. There are concerns too that children and young people will have been particularly negatively affected; it will be an important priority to restore children’s sense of security, emotional stability and opportunities for positive development [48] following the considerable disruption to education, peer and family relationships as a result of lockdown. WHO’s IASC has produced helpful guidance to highlight ways to include marginalised and vulnerable people during the emergency; this learning is relevant beyond the pandemic [29].

### Innovation

There are some good examples of how services and communities are responding in crisis that could inform the future development of services.

#### Recording COVID-19 experiences

The ‘Our Tomorrows’ story bank enables Kansas residents to share their experiences of COVID-19. Stories are shared to a state-wide portal and state-level decision-makers and community organisations have access to the Story Bank [49]. Analysis of the data informs interventions and provides a rapid response to local need. The European Federation of Psychologists’ Associations has stressed the importance of building a ‘community memory’ to help build resilience, be inclusive and establish learning to help respond to future challenges [50].

#### Telemedicine

An adolescent and young adult clinic in San Francisco responded to the emergency by replacing most in-person visits with telemedicine [51]. Young people found the service acceptable but common barriers were identified across clinical domains including the difficulty maintaining privacy and confidentiality. Using headphones, yes/no questions and the ‘chat’ function enabled patients to type replies to questions. Lower socioeconomic status compounded privacy challenges because of crowded living conditions and some professionals felt their clinical decision-making was limited by not being able to complete a physical examination or have access to laboratory data.

Elsewhere, technology has been scaled up. In Qatar, health clinics provide telephone/video medical consultations that allow patients to enter virtual consultations with their doctor with other specialists added where required. The system in turn can generate official electronic sick leave. Thirteen virtual clinics have been launched across primary care, secondary/tertiary care and include geriatric memory clinics, urgent primary care, mobile doctor clinics, mental health services for first responders, for children and adolescents, adults and older adults.

In Spain, mental health services had to adapt quickly. Where possible, people with severe conditions were moved to private clinics to ensure continuity of care. Local policy makers identified emergency psychiatry as an essential service to continue outpatient services. Home visits were organised for the most serious cases. IT experts enabled mental health staff working from home to access electronic clinical records while maintaining confidentiality [52].

The key themes from the scoping review have therefore included the importance of social inequalities; the accelerated development of new approaches to designing and delivering mental health support; and further highlighted the need for high quality data to inform how ongoing and evolving mental health needs can be most effectively addressed.

## Conclusions

The response to the emergency of COVID-19 has challenged and arguably permanently changed mental health services. This scoping review gives a snapshot of how the global community is trying to respond in a fast-changing environment and provides an overview which can inform the further policy and service responses that are needed. However services emerge from this crisis, many decision-makers are seeing this as an opportunity to make change, creating the potential to positively transform mental health care. There is consensus that this cannot be achieved without tackling the social determinants of health. Grave economic stressors add considerable threats to increase social inequalities and poverty. Countries also recognise that data collection, modelling and sharing needs to improve in order to inform planning. Specific at-risk groups need to be considered in service planning. Opportunities to build on positive elements emerging from crisis include the adaptability of community-based care; the importance of lived experience in services; improving interagency collaboration; appropriate use of digital healthcare; and connecting physical and mental health.

## Data Availability

Not applicable.

## Abbreviations

AI: Artificial intelligence
ARQ: ARQ National Psychotrauma Centre, the Netherlands
COVID-19: Coronavirus disease 2019
IASC: Inter-Agency Standing Committee
LMICs: Low- and middle-income countries
MHPSS: Mental health and psychosocial support
OECD: Organisation for Economic Co-operation and Development
UN: United Nations
